# IMM‐H007, a new therapeutic candidate for nonalcoholic fatty liver disease, improves hepatic steatosis in hamsters fed a high‐fat diet

**DOI:** 10.1002/2211-5463.12272

**Published:** 2017-08-29

**Authors:** Huijie Shi, Qingchun Wang, Liu Yang, Shouxia Xie, Haibo Zhu

**Affiliations:** ^1^ State Key Laboratory of Bioactive Substance and Function of Natural Medicines Beijing Key Laboratory of New Drug Mechanisms and Pharmacological Evaluation Study Institute of Materia Medica Chinese Academy of Medical Sciences & Peking Union Medical College Beijing China; ^2^ Department of Pharmacology Shenzhen People's Hospital Second Clinical College Jinan University Shenzhen China

**Keywords:** ^1^H‐NMR, 2′,3′,5′‐tri‐acetyl‐N6‐(3‐hydroxylaniline) adenosine, IMM‐H007, lipid metabolism, nonalcoholic fatty liver disease

## Abstract

Nonalcoholic fatty liver disease (NAFLD), the most common chronic liver disease in humans, is characterized by the accumulation of triacylglycerols (TGs) in hepatocytes. We tested whether 2′,3′,5′‐tri‐acetyl‐N6‐(3‐hydroxylaniline) adenosine (IMM‐H007) can eliminate hepatic steatosis in hamsters fed a high‐fat diet (HFD), as a model of NAFLD. Compared with HFD‐only controls, IMM‐H007 treatment significantly lowered serum levels of TG, total cholesterol, and free fatty acids (FFAs) in hamsters fed the HFD, with a prominent decrease in levels of serum transaminases and fasting insulin, without affecting fasting glucose levels. Moreover, ^1^H‐MRI and histopathological analyses revealed that hepatic lipid accumulation and fibrosis were improved by IMM‐H007 treatment. These changes were accompanied by improvement of insulin resistance and oxidative stress, and attenuation of inflammation. IMM‐H007 reduced expression of proteins involved in uptake of hepatic fatty acids and lipogenesis, and increased very low density lipoprotein secretion and expression of proteins responsible for fatty acid oxidation and autophagy. In studies *in vivo*, IMM‐H007 inhibited fatty acid import into hepatocytes and liver lipogenesis, and concomitantly stimulated fatty acid oxidation, autophagy, and export of hepatic lipids. These data suggest that IMM‐H007 resolves hepatic steatosis in HFD‐fed hamsters by the regulation of lipid metabolism. Thus, IMM‐H007 has therapeutic potential for NAFLD.

**Abbreviations**
ABCA1: ATP‐binding cassette subfamily A member 1
AMPK: 5′‐AMP‐activated protein kinase
HFD: high‐fat diet
NAFLD: nonalcoholic fatty liver disease
NASH: nonalcoholic steatohepatitis

Nonalcoholic fatty liver disease (NAFLD) is the most common cause of human liver disease worldwide and is considered to be the hepatic manifestation of the metabolic syndrome, which is closely associated with obesity, type 2 diabetes mellitus, and insulin resistance. NAFLD encompasses a broad spectrum of disease, ranging from simple steatosis with a benign prognosis to progressive nonalcoholic steatohepatitis (NASH) with inflammation and fibrosis, which can lead to cirrhosis and end‐stage liver disease. The prevalence of NAFLD has been estimated to be 20–30% in Western populations and 5–18% in Asian populations 1. Apart from modification of lifestyle and diet, no satisfactory therapy for NAFLD exists at present.

Lipid accumulation in hepatocytes occurs when the amount of fatty acid input exceeds the output, because of increased hepatocellular import of serum fatty acids, augmented *de novo* lipogenesis, reduced fatty acid oxidation and autophagy, and impaired export of triacylglycerols (TGs) from liver to blood 2. Therapies that regulate these processes and maintain a balance between the input and output of liver lipids may have important roles in the management of NAFLD.

IMM‐H007, also known as WS070117, is a novel lipid regulator with a structure that was discovered by investigators at the Institute of Materia Medica, Chinese Academy of Medical Science (CAMS), and Peking Union Medical College (PUMC). IMM‐H007 can regulate lipid metabolism in hamsters with hyperlipidemia and in the human hepatocellular carcinoma cell line HepG2 by activating 5′‐AMP‐activated protein kinase (AMPK), an important cellular energy sensor 3, 4. Treatment with IMM‐H007 promotes high‐density lipoprotein cholesterol efflux capacity and reverse cholesterol transport and attenuates atherogenesis by inhibiting degradation of ATP‐binding cassette subfamily A member 1 (ABCA1) protein or by reducing uptake of oxidized low‐density lipoprotein (LDL) 5, 6. However, the effects of IMM‐H007 on NAFLD have not previously been studied. In this study, our aims were to evaluate the effect of IMM‐H007 on NAFLD, and to explore the underlying mechanisms through which it acts.

## Materials and methods

### Chemicals

IMM‐H007 (WS070117), 99.7% pure (assessed by HPLC), was from the Institute of Materia Medica, CAMS, and PUMC (Beijing, China). Primary antibodies directed against lipoprotein lipase (LPL), microtubule‐associated proteins 1A/1B light chain 3B (LC3B), and adhesion G protein‐coupled receptor E1 (F4/80) were purchased from Abcam (Cambridge, UK). Primary antibodies directed against CD36, β‐actin, phosphorylated sterol regulatory element‐binding protein 1c (p‐SREBP‐1c), fatty acid synthase (FAS), stearoyl‐CoA desaturase (SCD1), Beclin‐1, sequestosome‐1 (p62), phosphatidylinositol 3‐kinase catalytic subunit type 3 (PI3K3C), RAC‐alpha serine/threonine protein kinase (AKT), p‐AKT, insulin receptor substrate 1 (IRS), p‐IRS, nuclear factor kappa light chain enhancer of activated B cells (NF‐κB), and p‐NF‐κB were purchased from Cell Signaling Technology (Danvers, MA, USA). Primary antibodies directed against NAD‐dependent protein deacetylase sirtuin‐1 (SIRT1) and peroxisome proliferator‐activated receptor γ coactivator 1‐α (PGC1α) were purchased from Santa Cruz Biotechnology (Santa Cruz, CA, USA). Carnitine O‐palmitoyltransferase 1, liver isoform (CPT1) antibody was purchased from Abgent (San Diego, CA, USA).

### Ethics statement

Animals used in this study were in accordance with the relevant federal guidelines and institutional policies, and the protocol was approved by the Animal Care and Use Committee at the Institute of Materia Medica, CAMS, and PUMC (Beijing, China). All surgeries were carried out under sodium pentobarbital anesthesia, and all efforts were made to minimize the animals’ discomfort.

### Study design

Syrian golden hamsters (6 weeks old, male, *n* = 50) were purchased from Vital River Lab Animal Technology (Beijing, China). The animals were kept in an environment with a constant temperature of 22 ± 1 °C, relative humidity of 55 ± 4%, and 12‐h light–dark cycle (08:00–20:00). They had free access to food and water. After 1 week of acclimatization, the animals were randomly divided into five groups: a normal control group with a chow diet, a model control group with a high‐fat diet (HFD), and three IMM‐H007 groups with HFD. Two weeks later, the IMM‐H007 groups were administered orally IMM‐H007 at 25, 50, and 100 mg·kg^−1^, twice a day (giving daily treatment levels of 50, 100, and 200 mg·kg^−1^). After 12 weeks of IMM‐H007 treatment, glucose and insulin tolerance tests were conducted and the TG secretion rate was measured. MRI examinations were carried out with a 7T Bruker BioSpec imaging system (Bruker, Billerica, MA, USA) equipped with a 16‐cm horizontal‐bore magnet. The animals were then sacrificed; serum was collected for biochemical analysis and livers for histopathological examination and immunoblotting.

### Glucose and insulin tolerance tests

In the glucose tolerance test, hamsters that had fasted for 16 h were injected intraperitoneally with 20% (w/v) glucose (2 g·kg^−1^ body weight) and blood samples were collected from the retrobulbar vein 0, 30, 60, 120, and 180 min later to determine serum glucose using the Accu‐Chek Performa system (Roche Diagnostics, Basel, Switzerland). In the insulin tolerance test, hamsters that had fasted for 2 h were injected with recombinant insulin (0.75 U·kg^−1^), blood samples were collected from the retrobulbar vein 0, 30, 60, 120, and 180 min later, and serum glucose was determined as previously described 7.

### Triacylglycerol secretion rate

The rate of TG secretion was measured as previously described 8. Briefly, the hamsters were injected intraperitoneally with poloxamer 407 (P407; Sigma, St. Louis, MO, USA; to inhibit hydrolysis by LPL) at 1000 mg·kg^−1^ after fasting for 4 h. Blood samples were collected immediately prior to injection and at 1, 2, 6, and 24 h following injection, and serum was taken to determine TG concentrations. The TG secretion rate, expressed in micromoles of TGs per kilogram body weight per hour (μmol·kg^−1^·h^−1^), was calculated from the difference in serum TG concentrations over a given interval after P407 injection.

### Measurement of serum biochemical markers

Serum levels of total cholesterol (TC), TGs, and free fatty acids (FFAs) were determined using commercially available enzymatic assay kits [Sekisui Medical Technology (China), Beijing, China]. Serum concentrations of aspartate aminotransferase (AST) and alanine aminotransferase (ALT) were analyzed using commercial clinical diagnosis kits (Nanjing Jiancheng Bioengineering Institute, Nanjing, China), with the manufacturer's standards and protocols.

### Liver histopathological evaluations

Liver tissues were trimmed (to 2 mm thickness) and then fixed in 4% neutral‐buffered formaldehyde, processed, and embedded in paraffin, and 5‐μm sections were stained individually with hematoxylin and eosin (HE; Baso, Zhuhai, China), Sirius Red (TIANDZ, Beijing, China), and Masson (Baso) to evaluate hepatic steatosis and fibrosis.

### Hepatic lipid measurements

Snap‐frozen liver tissues were homogenized and lysed in a buffer (50 mm Tris/HCl (pH 7.4), 150 mm NaCl, 1% (v/v) NP‐40, 0.1% (w/v) SDS), and centrifuged at 14 000 ***g*** for 10 min at 4 °C. Supernatants were collected, to enable determination of the concentrations of TC and TGs, using commercially available enzymatic kits [Sekisui Medical Technology (China)].

### Hepatic antioxidant capacity and lipid peroxidation

Snap‐frozen liver tissues were homogenized in ice‐cold phosphate‐buffered saline and centrifuged at 3500 ***g*** for 15 min at 4 °C. The supernatant was then collected and commercially available enzymatic kits were used to determine the concentrations of reduced glutathione (GSH; Nanjing Jiancheng Bioengineering Institute) and malondialdehyde (MDA; Beyotime Institute of Biotechnology, Jiangsu, China) and activities of superoxide dismutase (SOD; Beyotime Institute of Biotechnology), catalase and GSH peroxidase (GSH‐Px; Nanjing Jiancheng Bioengineering Institute).

### Hepatic levels of TNF‐α and IL‐6

Snap‐frozen liver tissues were homogenized and lysed in a buffer (50 mm Tris/HCl (pH 7.4), 150 mm NaCl, 1% (v/v) NP‐40, 0.1% (w/v) SDS), supplemented with complete protease inhibitors cocktail (Roche) and centrifuged at 12 000 ***g*** for 20 min at 4 °C. Supernatants were collected for the analysis of tumor necrosis factor alpha (TNF‐α) and interleukin (IL)‐6 using commercial ELISA kits (R&D systems, Minneapoli, USA and Bluegene, Shanghai, China).

### *In vivo* ^1^H magnetic resonance spectroscopy of liver

The levels of liver lipids were further measured by single‐voxel‐localized ^1^H MRS, with a 7T/16 US NMR spectrometer equipped with a 16‐cm horizontal‐bore magnet. Anesthesia was induced and maintained with isoflurane in oxygen at 3% and 1%, respectively, during acquisition. A pneumatic pillow (SA Inc., Stony Brook, NY, USA) was utilized to perform triggered respiratory gating, to obtain static images by limiting data acquisition to the end of expiration. Each hamster examined was placed in a cylindrical coil (60 mm inner diameter). Respiratory triggered 1_Localizer sequence was performed to confirm the positioning of the coil with the following parameters: repetition time (TR) = 25 ms, echo time (TE) = 2.7 ms, flip angle (FA) = 30°, slice thickness = 1 mm, data matrix = 256 × 256, field of view (FOV) = 60 × 60 mm, and number of repetitions (NEX) = 2. To avoid obvious blood vessel and subcutaneous fat contributions, T1_RARE (rapid acquisition with relaxation enhancement) imaging was used to guide the placement of the spectroscopic volume of interest (VOI) with the following parameters: TR = 500 ms, TE = 9 ms, echo spacing (ES) = 9 ms, rare factor (RF) = 4, slice thickness = 1.5 mm, data matrix = 256 × 256, FOV = 70 × 70 mm, and NEX = 8. Respiratory‐gated single‐voxel‐localized ^1^H‐MRS was accomplished using the point‐resolved spectroscopy (PRESS) sequence. The measurement parameters were as follows: TR = 2500 ms, TE = 16.168 ms, FA RF1 = 90°, FA RF2 = 180°, FA RF3 = 180°, points = 2048, and VOI = 4 × 4 × 4 cm^3^. Because the magnetic field homogenization is critical, local shimming was carried out until the line width of water reached < 50 Hz, conditioning the spectral resolution. Spectral data were processed using Topspin 6.0 (Bruker). The lipid indices were defined and calculated as shown in Table 1 9, 10.

**Table 1:** Indices of hepatic lipids and fatty acid composition from analysis of ^1^H MR spectra

	Index	Calculation
Lipid quantity	Lipid/(water + lipid) ratio	Int(lipids)/Int(lipids + water)
Fatty acid composition	PUI	I_diallylic_/(I_allylic_ + I_diallylic_ + I_methylene_ + I_methyl_)
	UI	I_methene_/(I_methene_ + I_allylic_ + I_methylene_ + I_methyl_)
	UIs (surrogate unsaturation index)	(I_allylic_ + I_diallylic_)/(I_allylic_ + I_diallylic_ + I_methylene_ + I_methyl_)
	SI	1–UIs

Water, 4.7 p.p.m.; methene (–CH=CH–), 5.3 p.p.m.; diallylic (=CH–CH_2_–CH=), 2.9 p.p.m.; allylic (–CH_2_–CH=CH–), 2.1 p.p.m.; methylene (–(CH_2_)_*n*_–), 1.3 p.p.m.; methyl, (–CH_3_)_,_ 0.9 p.p.m. Int(lipids), the integral of –(CH_2_)_*n*_–; Int (lipids + water), the integral of –(CH_2_)_*n*_– and water; I_methene_, the signal amplitude of the methene, I_diallylic_, the signal amplitude of the diallylic methylene peak; I_allylic_, the signal amplitude of allylic methylene; I_methylene_, the signal amplitude of bulk methylene; I_methyl_, the signal amplitude of terminal methyl peaks.

### Western blot analysis

Liver tissues (100 mg) were homogenized in RIPA lysis buffer (300 μL; Beyotime, Beijing, China) supplemented with protease inhibitor cocktail (Roche Diagnostics) and phosphatase inhibitors (Applygen, Beijing, China). Liver homogenates were centrifuged at 12 000 ***g*** for 20 min at 4 °C. Supernatants were collected and diluted with RIPA lysis buffer containing protease inhibitors and phosphatase inhibitors, and the protein concentration was quantified using Bicinchoninic Acid Protein Assay Kit (Macgene, Beijing, China). The proteins were separated by 10–12% SDS/PAGE, transferred to polyvinylidene fluoride membranes, and incubated with primary antibodies at 4 °C overnight, and then with appropriate HRP‐conjugated secondary antibodies for 1 h at room temperature. The immunoreactive proteins were detected by chemiluminescence (ECL Plus Western blotting detection system; GE Healthcare, Chicago, IL, USA) and analyzed with image j software (NIH, Bethesda, MD, USA). The internal control was parallel blotting of β‐actin.

### Statistical analysis

Data are presented as mean ± standard error of the mean (SEM). Student's *t*‐test (two‐tailed) was used to evaluate the differences between means of two groups, and ANOVA was used to analyze the differences among three or more groups with Tukey's or Sidak's post hoc testing. Statistical significance was assumed at *P* < 0.05 or *P* < 0.01.

## Results

### Serum biochemical markers

High‐fat diet‐fed hamsters showed a significant increase in serum TG, TC, and FFAs compared with chow‐fed hamsters (Table 2). Treatment with IMM‐H007 markedly reduced serum levels of TG, TC, and FFAs in hamsters fed with HFDs. In addition, IMM‐H007 treatment resulted in a significant decrease in serum levels of transaminases, compared with HFD‐only controls. No significant differences were observed in serum levels of glucose between the groups. HFDs induced a marked increase in serum insulin relative to chow diets, whereas HFD‐fed hamsters treated with IMM‐H007 had lower levels of serum insulin than HFD‐only controls.

**Table 2:** Serum biochemical parameters in hamsters from different experimental groups

Parameters	Chow diet	HFD	HFD + H007 (50)	HFD + H007 (100)	HFD + H007 (200)
Serum TG (mmol·L^−1^)	1.9 ± 0.05	14.5 ± 0.56**	5.2 ± 0.12††	4.8 ± 0.12††	3.3 ± 0.10††
Serum TC (mmol·L^−1^)	3.6 ± 0.04	15.7 ± 0.37**	10.2 ± 0.24††	10.1 ± 0.18††	9.2 ± 0.08††
Serum FFA (mmol·L^−1^)	2156.4 ± 33.77	4190.1 ± 42.97**	3567.9 ± 36.86††	3334.6 ± 29.61††	2658.6 ± 46.76††
ALT (U·L^−1^)	9.9 ± 0.40	87.7 ± 4.42**	21.8 ± 0.64††	19.8 ± 0.82††	9.7 ± 0.44††
AST (U·L^−1^)	10.9 ± 0.26	30.5 ± 0.74**	18.9 ± 0.41††	18.7 ± 0.60††	14.7 ± 0.40††
Glucose (mmol·L^−1^)	4.0 ± 0.04	4.0 ± 0.03	4.1 ± 0.06	4.0 ± 0.03	4.0 ± 0.03
Insulin (nmol·mL^−1^)	0.6 ± 0.02	3.9 ± 0.16**	2.0 ± 0.09†	1.2 ± 0.04††	0.8 ± 0.01††

*P < 0.05, **P < 0.01 with respect to chow diet group

^†^ *P* < 0.05, ^††^ *P* < 0.01 with respect to HFD group.

HFD + H007 (50), high‐fat diet + IMM‐H007 (50 mg·kg^−1^); HFD + H007 (100), high‐fat diet + IMM‐H007 (100 mg·kg^−1^); HFD + H007 (200), high‐fat diet + IMM‐H007 (200 mg·kg^−1^). Data are expressed as mean ± SEM, *n* = 10 per group.

### IMM‐H007 treatment improves hepatic steatosis and fibrosis

**Figure 1:**
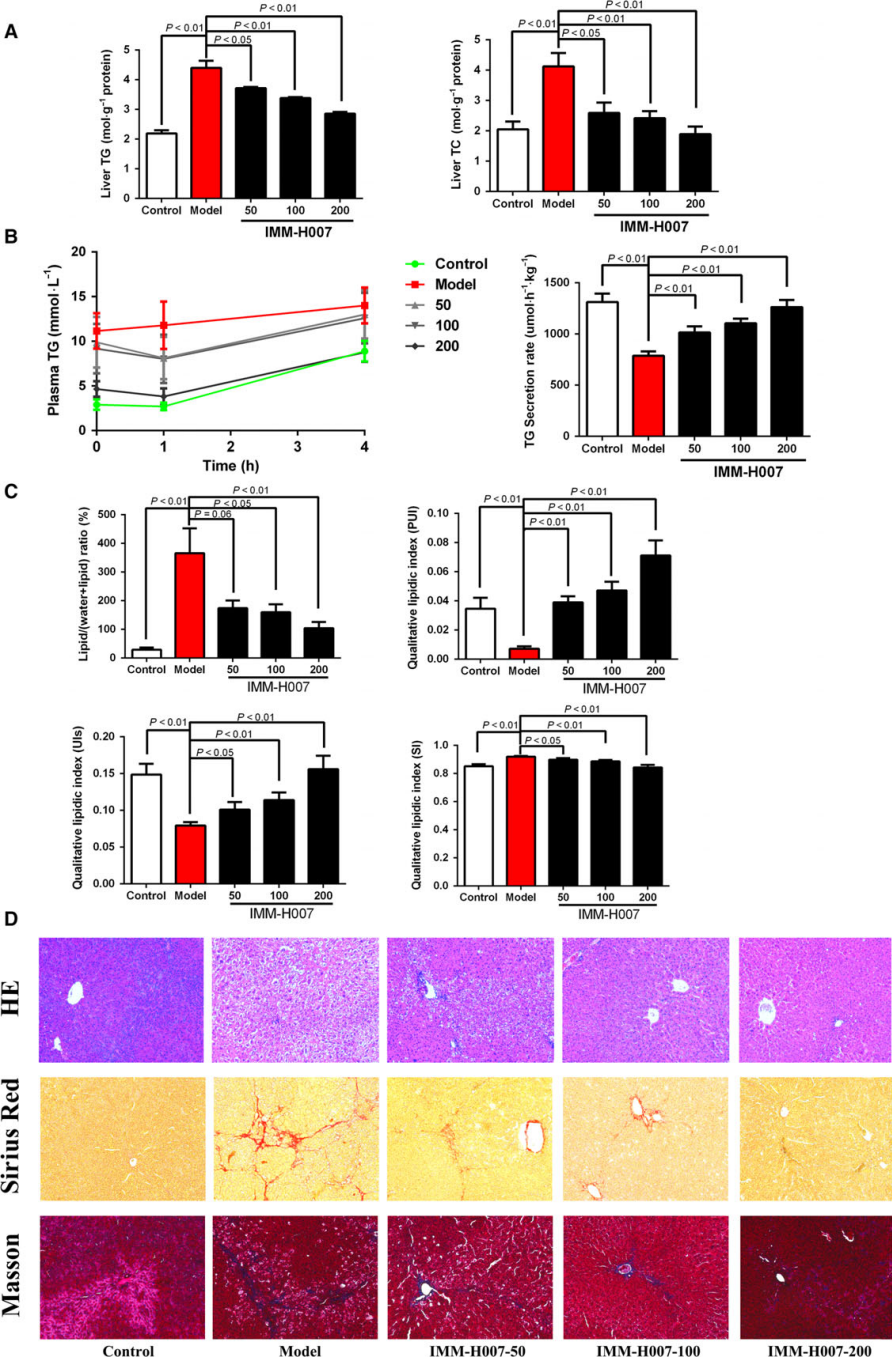
IMM‐H007 treatment decreases fat accumulation in HFD‐fed hamsters. (A). Biochemical analysis of liver samples. Levels of TC and TGs were measured in the supernatants of livers of hamsters in different experimental groups. (B). TG secretion rate (TGSR) from livers of hamsters in different experimental groups. (C). Quantitative and qualitative *in vivo* lipid assessments. The lipid/(water + lipid) ratio represents the lipid accumulation in the liver. PUI, unsaturated index surrogate (UIs), and SI reflect hepatic lipid composition. Data are expressed as mean ± SEM (*n* = 6–8 per group). (D). Hepatic histology, with staining by HE (X), Sirius Red, and Masson.

We investigated whether IMM‐H007 has an effect on HFD‐induced steatosis in hamsters fed with HFDs. Liver histopathological evaluations showed that HFD‐fed hamsters exhibited increased lipid accumulation in liver cells, whereas treatment of HFD‐fed hamsters with IMM‐H007 resulted in attenuation of steatosis (Fig. 1C). IMM‐H007 therapy improved hepatic fibrosis relative to HFD‐only hamsters, as determined by Sirius Red staining and Masson staining (Fig. 1C). In accordance with histopathological findings, HFD‐fed hamsters that received IMM‐H007 showed a marked reduction in the liver content of TGs and cholesterol, compared with HFD‐only controls.

Hepatocellular lipid accumulation can be quantified by the lipid/(water + lipid) ratio, which is the ratio of the integral of water [4.7 parts per million (p.p.m.)] to the integral of methylene and water (1.3 + 4.7 p.p.m.) 10. Significant increases in the lipid/(water + lipid) ratio were observed in HFD‐fed hamsters compared with chow‐fed hamsters. IMM‐H007 therapy induced a marked decrease compared with HFD‐only animals in lipid/(water + lipid) ratios, indicating that IMM‐H007 attenuated lipid accumulation in liver cells. In addition, *in vivo* MRS provided information about lipid composition. Compared with hamsters fed with chow diets, HFD‐fed hamsters showed significant decreases in the polyunsaturation index (PUI) and unsaturation index (UI), with concomitant increases in the saturation index (SI), suggesting a pathological balance of fatty acids consistent with a fatty liver. HFD‐fed hamsters administrated with IMM‐H007 had a significant increase in PUI and UI, accompanied by a significant decrease in SI, relative to HFD‐only controls (Fig. 1B). Taken together, these results show that HFD‐fed hamsters exhibit liver steatosis, which can be ameliorated by IMM‐H007 therapy.

### IMM‐H007 reduces liver fat import in HFD‐fed hamsters

**Figure 2:**
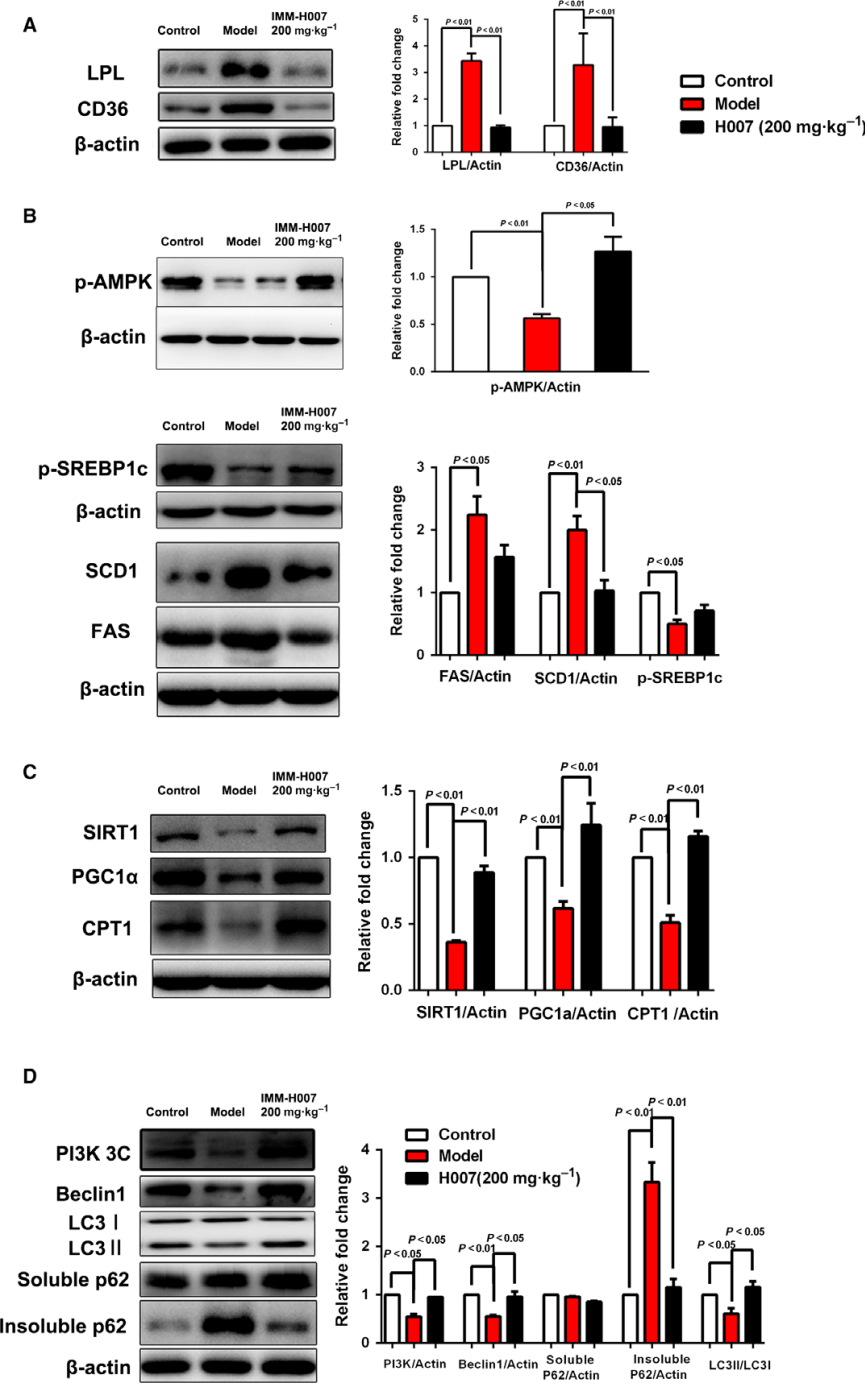
IMM‐H007 regulates hepatic lipid metabolism. (A). Western blots and densitometry to determine the levels of LPL and CD36, two important enzymes in hepatocyte uptake of fatty acids. (B). Western blots and densitometry to determine the levels of Ser372 phosphorylation of SREBP‐1c and Thr172 phosphorylation of AMPK, and levels of FAS and SCD1, in hamsters from different experimental groups. (C). Western blots and densitometry to determine the levels of SIRT1, PGC1α, and CPT1 in livers from hamsters in different experimental groups. (D). Western blots and densitometry to determine the levels of PI3K, Beclin‐1, LC3, and p62. Data are expressed as mean ± SEM (*n* = 6–8 per group).

In patients with NAFLD, about 60% of liver TGs are derived from nonesterified fatty acids and 15% from the diet 11. The import of fatty acids into hepatocytes involves two important molecules: hepatic LPL, which hydrolyzes circulating TGs, and fatty acid translocase CD36, which transports fatty acids across hepatocellular membranes. We observed significant downregulation of the levels of both LPL and CD36 in HFD‐fed hamsters treated with IMM‐H007, compared with HFD‐only controls, indicating that hepatocellular import of fatty acids was hampered by IMM‐H007 administration (Fig. 2A).

### Chronic treatment with IMM‐H007 decreases lipogenesis in livers of HFD‐fed hamsters

In addition to peripheral fatty acid flux, *de novo* lipogenesis accounts for 26% of liver content of TGs in patients with NAFLD. We investigated whether IMM‐H007 inhibits the process of lipogenesis. Two important enzymes are involved in hepatic *de novo* lipogenesis: FAS is responsible for fatty acid synthesis, thus contributing to hepatic steatosis, and SCD1 converts saturated fatty acids into monounsaturated fatty acids, facilitating the synthesis of TGs and other lipids. Hepatic expression of FAS and SCD1 was reduced significantly in hamsters treated with IMM‐H007, compared with HFD‐only animals (Fig. 2B).

SREBP‐1c is a key lipogenic transcription factor, which translocates to the nucleus, activates genes involved in the synthesis of fatty acids and TGs, and regulates the lipogenic process. However, when it is phosphorylated at Ser372 by activated AMPK, cleavage of the precursor decreases and nuclear translocation of SREBP‐1c is inhibited, resulting in improvement of steatosis 12. Consistent with the reduced expression of FAS and SCD1, levels of SREBP‐1c Ser372 phosphorylation were augmented in livers of HFD‐fed hamsters administered IMM‐H007 (compared with HFD‐only animals), with concomitant enhancement of AMPK activation (Fig. 2B).

### IMM‐H007 therapy affects hepatic fatty acid oxidation and autophagy in HFD‐fed hamsters

To further explore the mechanism by which IMM‐H007 ameliorates hepatic steatosis, we studied the effects of this small molecule on hepatic fatty acid oxidation and autophagy. Compared with HFD‐only controls, IMM‐H007 therapy increased hepatic expression of proteins promoting β‐oxidation, including SIRT1, PGC1α, and CPT1, which is the rate‐limiting enzyme of mitochondrial β‐oxidation (Fig. 2C).

To study the effect of IMM‐H007 on hepatic autophagy, the expression patterns of several molecular indicators were examined. Notably, compared with chow‐fed hamsters, HFD‐fed animals had reduced expression of PI3K3C and Beclin‐1, and decreased LC3B‐II : I ratios, with concomitant elevation of levels of insoluble p62 protein, but no change in the concentration of soluble p62. Compared with HFD‐only animals, IMM‐H007 treatment increased levels of PI3K3C and Beclin‐1 and LC3B‐II : I ratios, and reduced expression of insoluble p62 protein (Fig. 2D).

### IMM‐H007 treatment increases export of triacylglycerols from hepatocytes to blood

Impaired hepatic secretion of very low density lipoprotein (VLDL) can lead to hepatosteatosis, and therapies that can modulate this process could have a role in resolving hepatic steatosis. We examined whether IMM‐H007 can affect fatty acid export by cotreatment of animals with P407, an inhibitor of VLDL hydrolysis that does not affect lipoprotein metabolism 8. Compared with HFD‐only hamsters treated with P407, the VLDL secretion rate was higher in HFD‐fed hamsters treated daily with 200 mg·kg^−1^ IMM‐H007 and P407 (Fig. 1A).

### IMM‐H007 treatment affects hepatic inflammation and insulin resistance

**Figure 3:**
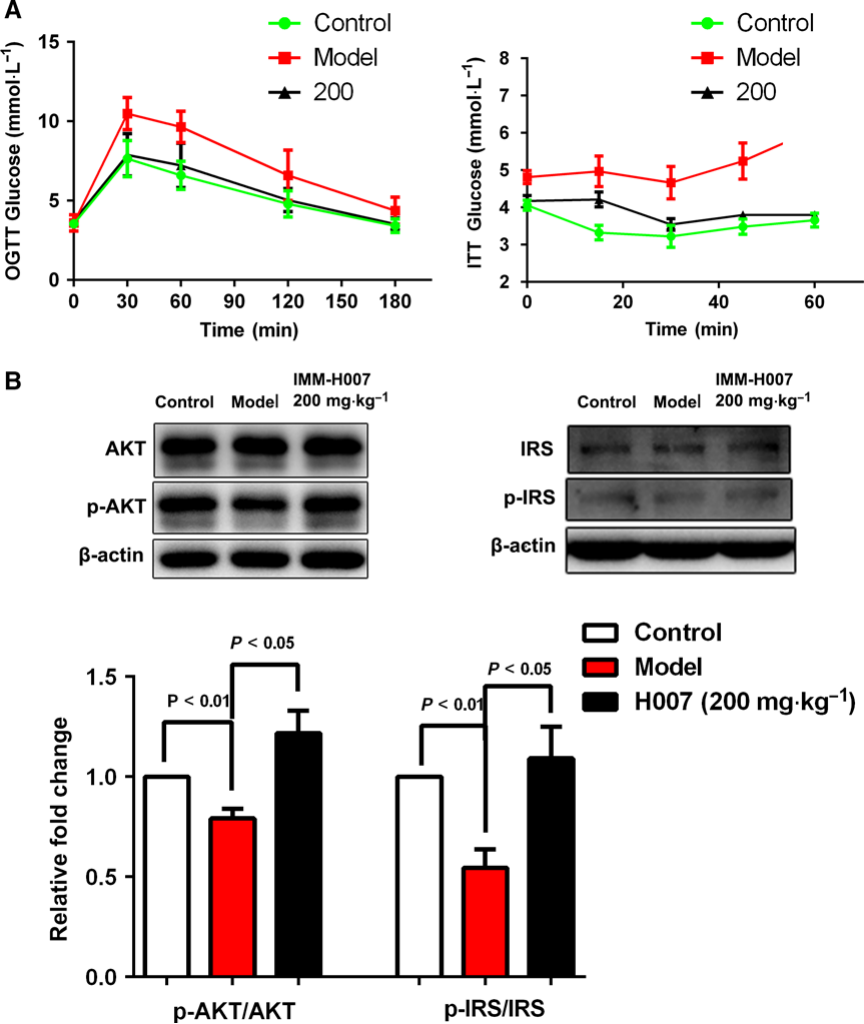
IMM‐H007 improves insulin resistance. (A). Glucose and insulin tolerance test results in hamsters from different experimental groups. (B). Western blots and densitometry to determine the levels of Ser473 phosphorylation of AKT and Tyr896 phosphorylation of IRS. Data are expressed as mean ± SEM (*n* = 6–8).

Consistent with our observation of decreased levels of serum transaminases, we found decreased levels of phosphorylated NF‐κB with no significant change in total levels of NF‐κB in HFD‐fed hamsters treated with IMM‐H007, compared with HFD‐only animals (Fig. 3A). Notably, HFD‐fed hamsters treated with IMM‐H007 exhibited significant downregulation of hepatic levels of the inflammatory cytokine tumor necrosis factor and IL‐6 (Table 3). Furthermore, IMM‐H007 treatment markedly reduced protein levels of F4/80, a specific macrophage marker, in HFD‐fed hamsters (Fig. 3A). These findings suggested that IMM‐H007 attenuates hepatic inflammatory reactions in HFD‐fed hamsters.

**Table 3:** Hepatic inflammatory factor content in hamsters from different experimental groups. HFD + H007 (200), high‐fat diet + IMM‐H007 (200 mg·kg^−1^). Data are expressed as mean ± SEM, *n* = 10 per group

Parameters	Chow diet	HFD	HFD + H007 (200)
TNF‐α (pg·μg^−1^ protein)	0.028 ± 0.019	0.23 ± 0.17^a^	0.045 ± 0.034^b^
IL‐6 (pg·μg^−1^ protein)	0.34 ± 0.09	0.94 ± 0.28^a^	0.31 ± 0.25^b^

a *P* < 0.01 with respect to chow diet group.

b *P* < 0.01 with respect to HFD group.

**Figure 4:**
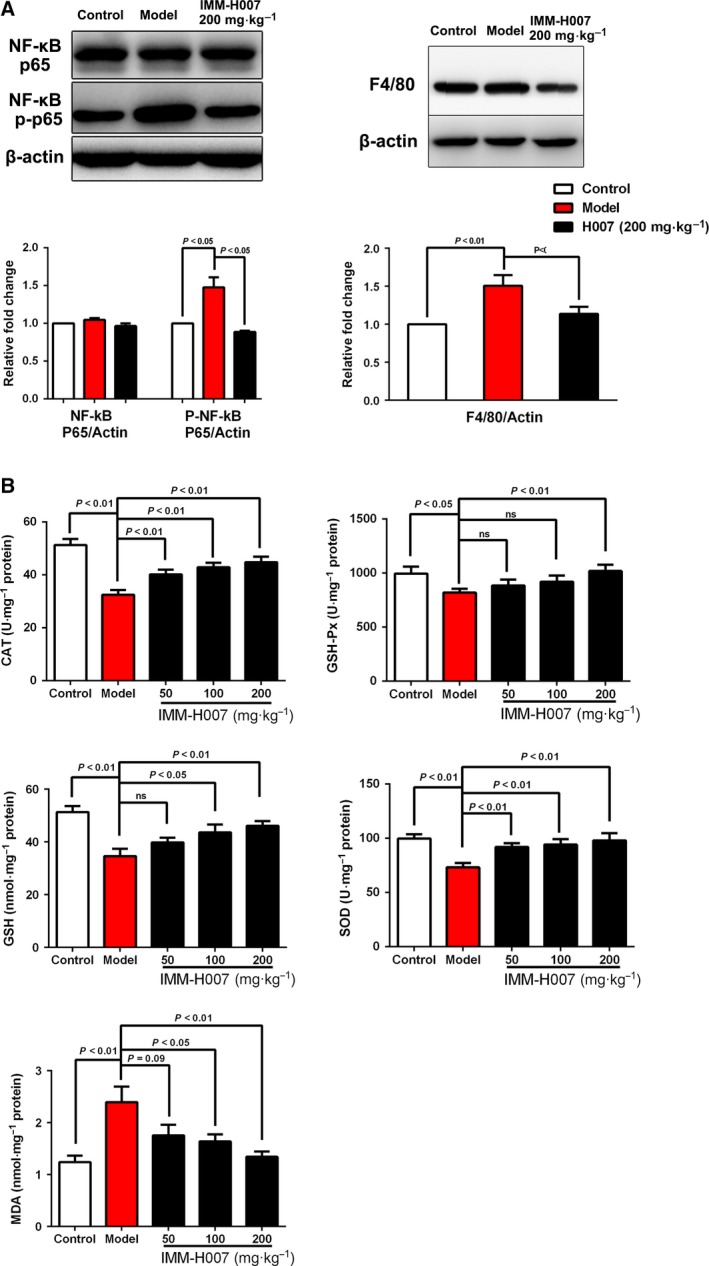
IMM‐H007 attenuates hepatic inflammation and improves oxidative stress in hamsters from different experimental groups. (A). Protein levels of NF‐κB, p‐NF‐κB, and F4/80 in livers from hamsters in different experimental groups. (B). Biochemical analysis of hepatic catalase (CAT), GSH‐Px, reduced GSH and SOD levels or activities, as well as MDA levels, in hamsters in different groups. Data are expressed as mean ± SEM (*n* = 6–10).

Insulin resistance is associated with the development of hepatic steatosis. In glucose tolerance tests, HFD‐fed hamsters treated with IMM‐H007 at a daily dose of 200 mg·kg^−1^ cleared glucose more effectively than HFD‐only hamsters. Moreover, in the insulin tolerance test, the response of HFD‐fed hamsters that received IMM‐H007 curves was close to that of chow‐fed hamsters (Fig. 4A). To further investigate the mechanisms underlying the response to IMM‐H007, phosphorylation of AKT and IRS was analyzed. Treatment with IMM‐H007 induced phosphorylation of AKT and IRS (Fig. 4B). These findings revealed that IMM‐H007 therapy improves hepatic insulin resistance.

### Chronic IMM‐H007 administration attenuates hepatic oxidative stress

Hepatic antioxidant capacity may be impaired by HFDs, resulting in oxidative stress, which is associated with the pathogenesis of liver disease 13, 14. We investigated the antioxidant effect of IMM‐H007 and found significant increases in hepatic activities of SOD, GSH‐Px, and catalase, as well as levels of reduced GSH, in HFD‐fed hamsters treated with IMM‐H007 (especially at the highest daily dose of 200 mg·kg^−1^), compared with HFD‐only animals (Fig. 3B). Levels of MDA, which is the final product of lipid peroxidation, were higher in HFD‐fed hamsters than in chow‐fed animals, and lower in hamsters that received IMM‐H007 than in HFD‐only controls.

## Discussion

In this study, we observed that the small molecule IMM‐H007 inhibits lipid accumulation in hepatocytes by reducing hepatic uptake of peripheral fatty acids, decreasing lipogenesis, enhancing fat consumption by fatty acid oxidation and autophagy of lipid droplets, and promoting export of hepatic TGs to the blood. Furthermore, IMM‐H007 attenuates hepatic inflammation, improves oxidative stress, and modifies hepatic insulin sensitivity, resulting in amelioration of NAFLD in HFD‐fed hamsters.

Hamsters fed HFDs for 12 weeks displayed many features of lipid metabolism resembling those of the metabolic syndrome in humans, with increased serum levels of TG, TC, and FFAs, accompanied by fasting hyperinsulinemia and an elevation of serum transaminases 15. However, whereas high fasting blood glucose is associated with the metabolic syndrome in humans, no significant change in levels of fasting glucose was observed in HFD‐fed animals. The dyslipidemia that was observed in HFD‐fed hamsters improved with IMM‐H007 treatment without affecting food consumption, as evidenced by reduced serum levels of TGs, cholesterol, and FFAs, together with decreased levels of plasma insulin and transaminases. The significant reduction in the plasma TG level after IMM‐H007 treatment suggests that plasma level of VLDL, which is determined by the rate of hepatic secretion and the rate of clearance from plasma, was also lowered. In this study, we found that IMM‐H007 significantly increased hepatic secretion of VLDL. Although the precise mechanisms need to be elucidated, we speculate that increased circulating VLDL clearance may account for the IMM‐H007‐induced improvement in the plasma TG level 16.

Noninvasive MRS is suitable for the quantification of liver fat and identification of the nature of the fat 17. ^1^H‐MRS analysis in HFD‐fed hamsters demonstrated that IMM‐H007 decreased the lipid/(water + lipid) ratio, reflecting reduced liver fat deposition, in accordance with biochemical data and the reduction in histologically determined steatosis, suggesting that MRS enables accurate quantification of liver fat. We also utilized MRS to identify modifications in the nature and proportions of fatty acids that are associated with steatosis 18, 19. The results indicated that HFD‐fed hamsters exhibit a significant increase in SI and a decrease in PUI (corresponding to increased hepatic TG content) compared with chow‐fed hamsters with normal hepatic TG content. IMM‐H007 treatment decreased SI and increased PUI relative to HFD‐only hamsters, and reduced the liver content of TGs. Taken together, our data reveal that MRS is a suitable tool for the quantification of liver lipid content and identification of lipid composition, and show that hepatic steatosis is closely associated with alterations of hepatic lipid composition. Our findings suggest that *in vivo* MRS may have applicability for the noninvasive monitoring of nutritional status in NAFLD patients with diseased livers.

Although exercise and caloric restriction are efficacious for the treatment of NAFLD, they have high dropout rates. Therefore, many pharmacologic strategies have been investigated for improving NAFLD, whose common characteristic is activation of AMPK. Cordycepin is an adenosine analog that is isolated from the fungus *Cordyceps militaris*. Previous studies found that cordycepin has beneficial effects on the liver *in vivo* 20, but its usefulness is limited by poor bioavailability. IMM‐H007 was derived from cordycepin and synthesized as a purine analog. IMM‐H007 activates AMPK and regulates lipid metabolism in hamsters and HepG2 cells 21. AMPK has an important role in the modulation of hepatic lipid metabolism that includes the suppression of lipogenesis and stimulation of fatty acid oxidation. Our results have now demonstrated enhancement of AMPK activation in hamsters subjected to chronic IMM‐H007 treatment. In this study, we investigated the effect of IMM‐H007 on *de novo* lipogenesis and fat consumption, including fat oxidation and autophagy.

Previous results demonstrated that SIRT1 activity is enhanced by AMPK, resulting in deacetylation and activation of PGC1α, which drives fatty acid oxidation and mitochondrial biogenesis 22. Overexpression of SIRT1 in mice attenuates hepatic steatosis and improves insulin sensitivity 23. Moreover, phosphorylation of PGC1α induced by AMPK facilitates PGC1α deacetylation by SIRT1 22. Accordingly, we observed increased expression of SIRT1 and PGC1α in response to treatment with IMM‐H007, in addition to enhanced AMPK activation. Expression of CPT1, a rate‐limiting enzyme in lipid oxidation, was elevated significantly, suggesting that IMM‐H007 therapy markedly increases fatty acid oxidation in livers of HFD‐fed hamsters and that this mechanism underlies the amelioration of hepatic steatosis by IMM‐H007.

IMM‐H007 activates fat disposal not only by stimulating fatty acid oxidation, but also by inducing autophagy, a lipolytic process in which portions of lipid droplets, or even whole droplets, are transported to lysosomes and degraded. Autophagy has an important role in the modulation of insulin resistance, and in lipid metabolism and improvements in NAFLD 24, 25. Beclin‐1 forms a complex with the class III PI3K (PI3K3C), and this complex mediates nucleation of the phagophore. The conjugation of LC3B to the membrane lipid phosphatidylethanolamine is mediated by ubiquitin‐like‐conjugating enzyme ATG3 and ubiquitin‐like modifier‐activating enzyme ATG7 to form LC3B‐II, which presents a recognition site for LC3B‐binding chaperones such as p62 to deliver their cargo to autophagosomes 26. We investigated autophagy markers and found that IMM‐H007 administration increased the expression levels of Beclin‐1 and PI3K, as well as the ratio of LC3B‐II : I, but decreased the level of insoluble p62 in HFD‐fed hamsters, indicating that IMM‐H007 does contribute to autophagy in HFD‐induced NAFLD.

Our results also demonstrated an effect of IMM‐H007 on liver TG synthesis and export of TGs from hepatocytes to blood through incorporation into VLDL, which contributes to resolution of steatosis in HFD‐fed hamsters. SREBP‐1c is an isoform of SREBP, a key lipogenic transcription factor; SREBP‐1c preferentially regulates lipogenic genes involved in the synthesis of fatty acids and TGs 27. SREBP‐1c has to be proteolytically processed to yield the active form, which then translocates into the nucleus, activating gene expression and promoting the lipogenic process in the liver. SREBP‐1c is phosphorylated by activated AMPK, resulting in decreased proteolytic cleavage of the protein, inhibition of nuclear translocation, and ultimately amelioration of hepatic steatosis 12. Accordingly, we found increased phosphorylation of SREBP‐1c in livers from IMM‐H007‐treated hamsters, compared with HFD‐only controls. This is also observed when resveratrol, a potent AMPK activator, is used. SCD1 and FAS are two key enzymes involved in lipogenesis 28, and the levels of these proteins were reduced by IMM‐H007 administration in HFD‐fed hamsters, suggesting inhibition of hepatic lipogenesis. Impaired VLDL secretion from hepatocytes to blood has been implicated in the pathogenesis of steatosis 29, 30. When we measured the secretion of VLDL in HFD‐fed hamsters chronically treated with IMM‐H007 and also given P407, an inhibitor of VLDL hydrolysis that does not affect lipoprotein metabolism, we found that IMM‐H007 treatment significantly increases the VLDL secretion rate, contributing to the improvement of hepatic steatosis.

Insulin resistance contributes to the initiation and progression of NAFLD 31. Our results demonstrated that IMM‐H007 ameliorates insulin resistance in HFD‐fed hamsters. Although IMM‐H007 did not affect the level of fasting glucose in this animal model, the serum level of insulin decreased markedly. In addition, data from glucose and insulin tolerance tests indicated that IMM‐H007 improves insulin sensitivity. IMM‐H007 therapy dramatically enhanced hepatic insulin signaling, as evidenced by increased phosphorylation of IRS and AKT in livers of HFD‐fed hamsters treated with IMM‐H007. The decrease in hepatic lipid content in these animals might account for the enhanced hepatic insulin sensitivity, although other mechanisms may also be involved, such as attenuation of inflammation and improvement of oxidative stress by IMM‐H007, in turn contributing to improved insulin resistance and steatosis.

AMPK activation by IMM‐H007 may partially explain the observed decrease in hepatic uptake of peripheral fatty acids and lipogenesis, the increase in fatty acid oxidation and autophagy of lipid droplets, and the promotion of hepatic TG export to the blood, because AMPK activation inhibits hepatic uptake of fatty acids and *de novo* lipogenesis, and increases fat consumption and fat export from the liver. However, previous studies have shown that the leptin, adiponectin, and PPAR ligands have roles in the amelioration of liver steatosis 32, 33, 34. Whether AMPK activation is the only way that IMM‐H007 improves liver steatosis or whether other processes also participate warrants investigation.

In conclusion, IMM‐H007 treatment in HFD‐fed hamsters improved steatosis by the regulation of lipid homeostasis in the liver, improvement of insulin resistance and oxidative stress, and attenuation of inflammation. These results demonstrate the potential of IMM‐H007 as an effective therapy for amelioration of NAFLD.

## Author contributions

HS and QW designed and conducted experiments, analyzed data, and wrote the manuscript. HZ designed experiments, wrote the manuscript, and supervised this project. LY applied for permission from the institutional review board of Materia Medica, CAMS, and PUMC (Beijing, China). SX supervised the work presented in this manuscript.
